# The Impact of Infertility on Daily Occupations and Roles

**Published:** 2019

**Authors:** Megan Edwards Collins

**Affiliations:** Occupational Therapy Department, Winston-Salem State University, Winston-Salem, USA

**Keywords:** Infertility, Roles, Self-care

## Abstract

**Background:**

Infertility impacts approximately 16% of couples in the United States-roughly five million individuals. Experiencing infertility takes a physical and psychological toll on the infertile individual, as well as his/her partner. The goal of the current study was to explore and provide better insight into how infertility affects the roles and daily occupations (Such as self-care and work related tasks) of females. This information can better assist health care providers in providing quality care to such clients.

**Methods:**

21 participants, females ranging in age from 20 to 46 years and experiencing infertility, participated in this qualitative, phenomenological research study. They partook in two telephone interviews aimed at exploring how infertility has impacted their roles and daily occupations. Inductive content data analysis was utilized to analyze the data.

**Results:**

Findings resulted in three main themes. Quotes from participants were used to title the themes. They are “when you’re dealing with infertility, every aspect of your life is impacted by it”, “infertility impacted my areas of interest in life” and “infertility is very lonely”.

**Conclusion:**

Infertility has the potential to impact every area of a female’s life. The emotional impact infertility may have on women, in addition to the physical and time constraints involved with pursuing fertility treatments, frequently resulted in decreased occupational engagement and fulfillment of roles as spouse or friend. Those experiencing infertility need more resources and support to navigate their journey.

## Introduction

Infertile couple defined as having trouble conceiving a child after actively trying for at least 12 months, infertility impacts approximately 16% of couples in the United States-roughly five million individuals (1, 2). Many couples desire to have a child (3), as raising a family is often a cultural expectation and human desire (2). Discovering that one cannot have a child can be a devastating event. Experiencing infertility takes a physical and psychological toll on the infertile individual, as well as his/her partner (3, 4).

Research on infertility has found that feelings of stress, anger, guilt, depression, grief, anxiety, withdrawal, decreased self-esteem, loss of relationships, and decreased financial security (*e.g*., perhaps as a result of paying for medical interventions) may result (2–5). This includes questioning one’s worthiness as a potential parent and as a spouse, and experiencing a sense of loss of control over one’s life (2). Relationships with friends, family members, and spouses may be strained as those dealing with infertility may feel misunderstood or uncomfortable in situations, such as baby showers, where they are reminded of their infertility (1, 2). Changes in lifestyle, such as decreased leisure activities, may also result for various reasons (*e.g*., emotional stress or having decreased time for leisure activities due to time spent pursuing fertility treatments) (1). Furthermore, individuals dealing with infertility might feel a sense of loss over the dream family and life they had in mind for themselves (1, 5). Overall, research has shown that approximately half of women experiencing infertility rated it as the most stressful experience of their life, and 18% of couples reported that infertility has had a negative impact on their marriage (2).

There is limited research exploring how infertility may impact an individual’s daily roles and occupations. The focus of qualitative studies is often on the psychological effect of infertility, with daily changes in roles and routines not being mentioned or only briefly discussed in findings sections (2, 3, 5). Given the stress and changes an individual experiencing infertility may encounter, the current research study aimed to explore how infertility can alter an individual’s roles and occupations such as self-care and leisure activities, an area lacking in the literature. While the emotional impact of infertility has been explored in previous research, how one’s daily roles and activities are impacted has not thoroughly been addressed.

The American Occupational Therapy Association’s definitions of occupations and roles were used for this study. The American Occupational Therapy Association (6) defines occupations as “daily life activities in which people engage” (p. S43). This includes activities of daily living (*e.g*., dressing, bathing, toileting), instrumental activities of daily living (*e.g*., cooking, household management, driving), educational and work pursuits, play and leisure activities, and social participation. It defines roles as “sets of behaviors expected by society and shaped by culture and context that may be further conceptualized and defined by the client” (p. S45). Roles can include that of a parent/spouse, daughter/son, friend, and employee.

Exploring how roles and occupations are impacted by infertility can best be accomplished by taking a qualitative, phenomenological approach. This provides the opportunity for participants to share firsthand what their experiences with infertility have been like and to express their perspectives (7).

## Methods

The current study took a qualitative, transcendental phenomenological approach (8). A phenomenological study seeks to understand how a phenomenon is experienced by the majority of individuals, including the meaning they attribute to the experience. The findings aim to help others understand how the experience is understood by the majority and the main components involved in the phenomenon (7).

The phenomenon being explored was the experience of infertility, specifically how it impacts the roles and occupations of women. Once Institutional Review Board approval was obtained, the principal investigator emailed a flyer with information about the study to local infertility support groups and administrators of social media and informational sites related to infertility. In a snowball effect, some participants also provided the researcher’s contact information to friends who they thought might be interested in participating.

When contacted by an individual interested in participating in the study, the principal investigator determined that she met all eligibility requirements and was willing to complete a Role Checklist and participate in two recorded phone interviews. All potential participants who contacted the researcher and met the eligibility requirements were included in the study. Eligibility requirements included being female, between 18 and 45 years old, and having received a diagnosis of infertility by a medical professional. The Role Checklist is a self-administered measure with satisfactory construct validity and content face validity and adequate test-retest reliability (9). The Role Checklist obtains information about a person’s occupational roles and the value that individual places on the roles. For example, roles listed on the Role Checklist include Volunteer, Friend, and Home Maintenance. Individuals filling out the Role Checklist indicate if this is something they have done in the past, present, or plan to do in the future. They then indicate whether the feel or the role is not at all valuable, somewhat valuable, or very valuable. The Role Checklist was included in the study to get an idea of what occupations and roles the participants had. Participants completed the Role Checklist and signed an informed consent form prior to their interview. Both were emailed to the primary researcher and then reviewed during the interviews to ensure participant understanding and to address any questions. The standardized, open-ended interviews were audio recorded and then transcribed by a graduate research assistant.

Basic demographic information (Such as age and ethnicity) and background medical information (Such as how long the participant has been trying to conceive) was obtained initially. While interviews were informal, basic questions were asked of all participants. They included “What was the experience of being diagnosed with infertility like?” “How do you feel infertility has impacted your daily life?” “How do you feel infertility has impacted your role as a … (wife, sister, aunt)?” “Is infertility something you openly speak about with friends and family?” “For you, what has been the hardest thing about being diagnosed with infertility?” and “How can occupational therapy practitioners better support you?”. Individual responses to the Role Checklist were used to guide interview questions (*e.g*., if the participant indicated on the checklist that they worked then the researcher asked questions related to work). Interviews lasted on average between thirty and sixty minutes. The researcher was prepared to guide participants to resources if needed, to address ethical considerations such as emotional distress participants might experience given the sensitive topic being explored.

Once an interview was transcribed, the primary researcher analyzed the data using inductive content analysis. Themes were derived based on their relevance to the research question of how infertility impacts an individual’s roles and occupations, and how occupational therapy practitioners can better support these individuals. The process included reading interview transcripts and highlighting the major concepts and ideas being conveyed. The resulting concepts were continually reviewed and grouped together to form themes related to the research question. Redundant information, or information specific to an individual case, was eliminated.

Once a participant’s initial interview was analyzed, a follow-up interview was arranged. These interviews were conducted 1–2 months after the participant’s initial interview, and lasted approximately 30 *min*. At this time, as a form of member checking, feedback was sought on themes the principal investigator found, and the principal investigator sought clarification on areas that were unclear from the initial interview. Any additional information the participant wished to add was also obtained. Follow-up interviews were audio recorded, transcribed, and analyzed like the initial interviews, and then combined with the participant’s initial interview. Once the data from all participants was analyzed, themes were combined across participants to develop final themes and subthemes. These included themes that the majority of participants experienced and reported, and that were relevant to the study’s aims and objectives. The graduate research assistant who transcribed the data independently analyzed the interviews, and the researcher compared the themes of the graduate research assistant with her own themes to increase the rigor and validity of the results. Data saturation was reached, where information from new participants either reinforced what had already been disclosed by previous participants, or new information was related to a participant’s particular situation.

It is important to note that the author has had personal experience with infertility. To ensure the integrity of the results and to assist in decreasing her personal biases as much as possible, two steps were taken. First, the author’s graduate research assistant independently reviewed and analyzed the data and then the author’s finding and the graduate research assistant’s findings were compared. This included both the researcher and the graduate assistant reviewing the analysis the other conducted, and discussing any discrepancies. Data was reviewed again as needed to ensure changes made as a result of the discussions reflected what the participants disclosed in interviews. Secondly, the follow-up interviews included the researcher reviewing the themes from the participant’s first interview and seeking feedback to make sure that the analysis that had been conducted accurately captured the essence of the participant’s experience. Changes were made as needed based on participant feedback to ensure accuracy and to highlight the most pertinent information. Furthermore, the author engaged in the qualitative strategy of Epoche, also called bracketing in some sources (LIN). Epoche relates to steps taken to better ensure that the findings were derived from research participants and were not impacted by the personal thoughts, feelings, and experiences of the researcher (s). In the current study, the author frequently reflected and journaled the emotions she was feeling so she was able to separate (E.g., bracket) her biases and better ensure they were not impacting the findings (10). Throughout the study and data analysis process, the author was aware of her personal opinions and tried to disregard her own beliefs and values while analyzing the data. This was done in an effort to prevent her own experiences and opinions from impacting the study’s findings.

## Results

### Participant demographics:

In all, 21 women between the ages of 20 and 46 experiencing infertility, or who had experienced infertility in the past, participated in the current study. Located all across the United States, including, California, Florida, South Carolina, Wisconsin, Montana, Washington, Colorado, New York, and Pennsylvania, their average age was 29.8 years old. A 46 years old participant was included as she experienced infertility when she was younger. One participant was also from Ontario, Canada. Further information about each participant is included in table 1. Reasons for infertility included Turner Syndrome, Uterine Polyps, Polycystic Ovarian Syndrome, Endometriosis, and Ovarian Failure. Six participants experienced infertility due to medical complications their male counterpart experienced. These included decreased sperm quality and mobility. Of note, two participants became pregnant in-between their first and second interview. One of these contacted the primary researcher after her final interview to let her know she had had a miscarriage. Participant demographics are shown in table 1.

**Table 1.** Participant demographics

**No**	**Age (years)**	**Race**	**Employment**	**Marital status**
**1**	20	Caucasian	Undergraduate student	Single
**2**	39	Hispanic, Caucasian	Adjunct faculty	Single
**3**	31	Caucasian	Graduate student	Single
**4**	44	Caucasian	Vice president of sales	Long-term relationship
**5**	32	Middle Eastern and European	Marketing company	Married
**6**	37	Caucasian	Principal	Married
**7**	35	Caucasian	Medical resident	Married
**8**	36	Caucasian, Latino	N/A	Married
**9**	27	Caucasian	N/A	Married
**10**	33	Caucasian	Accountant	Married
**11**	42	Caucasian	Human resources	Married
**12**	36	Caucasian	Foster care company	Married
**13**	34	Caucasian	Research	Married
**14**	36	Caucasian	Pharmaceutical company	Married
**15**	38	Caucasian	Attorney	Married
**16**	33	Caucasian	Post-doctoral scholar	Married
**17**	28	Caucasian	Occupational therapist	Married
**18**	32	Caucasian	Graduate student	Married
**19**	46	Caucasian	Chiropractor	Married
**20**	30	Caucasian	Business analyst	Married
**21**	27	Caucasian	Graduate student	Married

### Findings:

Findings resulted in three main themes. Quotes from participants were used to title the themes. They are “when you’re dealing with infertility, every aspect of your life is impacted by it” (Subthemes of fertility treatments and survival and self-preservation), “infertility impacted my areas of interest in life” (Subthemes of self-care and leisure and career), and “infertility is very lonely” (Subthemes of secrecy and misunderstood). These themes and subthemes reflect the experiences and sentiments of majority of participants.

“when you’re dealing with infertility, every aspect of your life is impacted by it” (Participant five) (Subthemes: Fertility treatments and survival and self-preservation)

While a few participants reported that experiencing infertility had no influence on their occupations, the majority felt it impacted all areas of their life. In a statement that summarizes the sentiment of most participants, participant 5 reported that, “when you’re dealing with infertility, every aspect of your life is impacted by it, whether it be small or large scale”. Similarly, participant 10 stated that “it takes over your life, it is always in your mind”, while participant 11 stated that, “it’s constant in your thoughts because you don’t have a choice”. Participant 3 went on to discuss how infertility “does … effect your vision for your overall life. … What you see happening in your life. What your expectations might be. … What you expect your life to be like”. Participant 5 stated, “I think it has made life harder on me”.

Furthermore, Participant 12 stated that: “I think so much of who we are identified as the woman aspect is our ability to have children. So, when not having that ability or not being able to, you feel like you are broken. It is supposed to be something that everyone can do. Your body is made for it and you can’t do it. I think that is where a lot of the struggle or difficulty comes from. It’s hard to connect with other people as well when you are thinking about it day to day, every day, of your life”.

Managing fertility treatments and the emotional impact of infertility greatly impacted the daily life and roles of participants.

### Fertility treatments:

Fertility treatments were noted by participants to be time consuming, unpredictable, and costly; making pursuing fertility treatments a challenge to moving on with daily life. Participant 14 noted that “my daily life has changed a lot, especially when you are undergoing treatments”. Similarly, participant 19 stated that: “It impacted my daily life in that I pretty much completely shut down. Other than self care, I just withdrew. I would get up, go run, shower, get to work, and talk to everyone on a superficial level. I could smile and fake my way through it for the most part”.

Some participants even reported that infertility had minimal impact when they were not undergoing treatments but a major impact during fertility treatments. Participants found that fertility treatments and navigating appointments were big time commitments, requiring them to balance treatments with work responsibilities and leisure engagement. Many times participants decreased or no longer engaged in leisure activities, hobbies, or self-care because of a lack of time. They also noted that appointments for treatments were often unpredictable, including finding out about an appointment, the day of the appointment. With frequent and variable doctors’ appointments, participants often felt like they were living appointment to appointment. As participant 10 reported, “you are waiting and don’t know what is going to happen. You can’t plan anything”. Participant 4 noted that, “There are so many things that are in your control, and this happens to be one out of my control; and it’s not a fun place to be”.

Furthermore, undergoing infertility treatment or other options for becoming a parent can be a huge financial commitment. Some participants noted that the extraordinary costs can be terrifying. In fact, a few participants reported that they had gone through their entire savings while pursuing fertility treatments. They noted that the financial cost of treatments can require creating a detailed budget that outlines how money will be handled and where it will be spent. This led to some participants having to make difficult decisions, impacting the occupations they engage in. For example, one participant noted that her husband and she had to decide whether to put money towards continuing education classes or fertility treatments. Participants also felt guilty when spending money on things other than treatments. As a result, many stopped taking vacations or engaging in other activities, like date night, in an effort to save money. Many felt like participant 17, who stated, “we need that money to be able to fund IVF cycles”. Participant 14 explained that such experiences lead to a “general feeling of lack of control. … I’m always going somewhere, there is always something to do. It becomes all-consuming in your life”. Making such decisions and undergoing fertility treatments led to many participants feeling like their lives were in a holding pattern.

Finally, pursuing fertility treatments was noted by participants to alter their marriage or partnership by adding tension and strain on the relationship. For example, some participants noted becoming angry, annoyed, agitated, and resentful towards their husbands if they were the medical reason for the infertility. This could be because participants often had to do more than their husbands did when pursuing infertility interventions, such as taking medications and undergoing procedures, even if the partner was the main reason behind the fertility challenges. Many echoed the sentiments of participant 16, who stated, “I was really frustrated when they told me I would have to go on fertility medications; take pills or injections to conceive. They said there was nothing wrong with me, so why should I have to do this?”.

Some also found that their husbands were laid back about having children and not actively engaging in activities that could assist in achieving pregnancy, leading to feelings of frustration and anger. This was the case with participant 21, who noted that “I think sometimes I resent him. There are times he has to do things for the process and he is last minute and lackadaisical about it. It makes me really annoyed”. The treatments and resulting stress and strain also impacted physical intimacy, where engaging in sexual relations became a job rather than an expression of love. Participant 12 summarized the sentiments of many participants when stating that: “It’s been rough. I think, as a woman or a wife, your role is to carry children for your family. I have outwardly said that there is something wrong with me, or what is my purpose if I can’t have children. Luckily, my husband says I’m crazy and that is not true. It doesn’t detract from being a caregiver, but it does question part of the duty of a wife or of a couple. It does make intimacy a little different, when you have been trying for so long and it isn’t working. It does make you try to figure out what time to have sex, or different things to try. It does create another level of craziness that normal couples don’t experience”.

Similarly, participant 17 noted that: “It’s challenging because it creates decisions we never would have made before, and adds stress and anxiety in a different angle to the relationship. Also … we have a lot of couple friends with kids, so we are constantly around kids. And sometimes we won’t as a couple go to events that are challenging or we know that there will be that element of grief. Or where you leave and you don’t feel emotionally fulfilled”.

### Survival and self-preservation:

The majority of participants reported that their infertility struggles were a constant thought. They found that these thoughts were all consuming, permeating their daily life and making it hard to concentrate on anything else. They felt that, when one experiences infertility, it is always on your mind, taking over your life and turning it upside down. These thoughts resulted in many participants “functioning less”, as they shut down and withdrew from activities and others. Frequently in need of “emotional caretaking”, they found themselves in survival and self-presentation mode. Participant 19 reported that, “It [infertility] effects daily functioning because you get depressed and anxious. You don’t want to go to work, have relationships, see other people who are pregnant”. Participants noted that there are often triggers that cause emotions related to infertility to surface. These can include being with friends who are pregnant or who have kids, seeing pregnant women or women with children, or seeing pregnancy and child-related things on television or social media. These triggers can cause a downward spiral, awakening negative emotions and thoughts that cannot be controlled. This can include feeling jealous of and bitter towards friends or siblings who have children. Participant 14 noted that “sometimes you have to be more guarded about things, how you react to things than maybe you would be otherwise”. Similarly, participant 19 reported that, “it was all about survival and self-preservation for me”.

Many participants deemed it unfair that others, who were not taking care of themselves, were getting pregnant while they were making major lifestyle changes to increase their chances of becoming pregnant. For example, participant 15 noted that: “That part has been hard [friends getting pregnant]. I feel like I take better care of myself than any of them do. It seems unfair and frustrating. I quit my job, changed my diet, and am super health conscious. And it seems like everyone else gets pregnant no problem. So that part emotionally has been hard”.

Participant 19 goes on to explain that, when others get pregnant, “It’s just brutal. And no one can understand how hard it is. You just feel horrible for having these feelings”. As it can be emotionally difficult to be around children and pregnancy related things, participants often distanced themselves from others, shutting out and avoiding friends, family, and anything associated with pregnancy. This included nieces/nephews and events with children such as birthday parties and baby showers. Participants did this as a way to put up boundaries and guard themselves from emotional triggers, which involved decreasing their conversations and interactions with others as they might not want to talk about their infertility struggles. Participant 17 summarized the feelings of many when she stated: “Ever since being diagnosed with infertility, it’s really challenging to be around kids. There is the rational me who knows that being with children meets the pseudo-need for me to have my own, but I can’t. An environment that used to be very emotionally fulfilling is now very emotionally draining”.

“infertility impacted my areas of interest in life” (Participant one) (Subthemes: Self-care and leisure and career)

Many participants noted that they often had to reprioritize things in their lives and change their focus so they could have more balance and decrease their stress. This frequently changed the participants’ perspective of their life path and who they are as individuals, altering their occupational engagement. For example, one participated noted that she and her husband have engaged in more community service activities to give back to others as a result of their infertility struggles. The specific impact experiencing infertility has on self-care tasks, leisure, and career pursuits was discussed by participants. Participant 1 reported that infertility, “definitely … impacted my areas of interest in life”.

### Self-care and leisure:

Many participants found they did not put as much effort into or have as much interest in self-care activities such as bathing. They found it took an increased effort to complete self-care tasks, that they were not cooking or cleaning as much, and that time commitments for appointments related to infertility made it hard to maintain a household and cook. As participant 17 noted, “the time available for me to do self-care has diminished”. Furthermore, painful and fatiguing treatments limited the physical ability of many participants to engage in self-care tasks for periods of time. Participant 9 reported that “for a little while I didn’t feel the need to put effort into it [self-care]”. Participant 12 noted, when discussing daily tasks like cooking and doing her hair, that “I just didn’t care. I don’t have the energy to move, so those things were the last of my list to do”. On the flip-side, some participants found they made changes in their self-care to be more health conscious and healthy in an effort to increase their chances of becoming pregnant. This included watching their overall diet, eating organic foods, going on a dairy/gluten free diet, cutting out alcohol and caffeine, engaging in exercise in an effort to lose weight, taking vitamins and supplements, quitting smoking, and seeing acupuncturists and nutritionists. For example, participant 19 noted that “it made me take greater self-care. I had to. I felt like I was in survival mode. I let myself do what I needed”.

While some engaged in leisure and hobbies as a form of emotional self-care, many participants felt that their hobby and leisure activities were negatively impacted. Participant 7 reported that, “it pretty much obliterated my leisure/hobby pursuits. All extra stuff. … It did take away any leisure time we did have”. They had to forgo meaningful and valued leisure/hobby pursuits, such as traveling, running, and taking vacations. This was emotionally challenging. For example, one participant was told by her physician to stop running. This devastated the participant, who enjoyed running and used it as a way to relieve anxiety and guilt. Not being able to run left her with no “outlet” for her emotions. Finally, during treatments, many participants reported that they were physically unable to engage in leisure/hobby activities due to pain. Participant summarized the sentiments of many participants when she stated that: “The reality is that it impacts so many different parts of your life. Like if you enjoy exercising … a lot of that you can’t do at certain times. And we have been doing this for so long it is a more constant thing”.

### Career:

Infertility altered the career path of a few participants, sometimes increasing their passion to work with kids and sometimes changing it so they could avoid being around children. A few participants were like participant 18, who stated that her “career choice and performance is influenced by me trying to mother everyone. You consider everyone as your child to take care of”. Many other participants found it difficult to concentrate on their careers, as they wanted to focus on having children. Working also made it hard to schedule appointments for fertility appointments and treatments, as participants had to get time off from work and in some cases obtain work coverage. Some noted that infertility challenges “totally disrupted it in every way” (Participant 21), while others found that not having children enabled them to achieve more at work because they did not have responsibilities related to children. This was expressed by participant 20, who stated If I don’t have kids, I can work more. I don’t have to worry about who is at home. It’s very good because I don’t have any responsibilities. That part is good now. If I have it [a child], I might have to stop working.

Emotionally, work gave some participants a sense of purpose while others found it stressful. Participant 4 noted that “work is actually the way to forget about it”, while participant 6 noted that “going to work was always good news, and feeling productive and successful, so that’s where I put my energy”. Other participants found managing a career while dealing with infertility stressful. Participant 17 echoed the feelings of many when stating that, “it’s hard juggling two areas. I can’t put my job down. I can’t push pause on the fertility treatment”, while participant 19 stated “you have to go to work, which adds stress”. Stresses noted by participants included managing medication side effects while at work, worrying about the perception employers might have of their performance if they learn about infertility treatments, and time management challenges (Including missing work for treatments or due to the side effects of treatments). The fact that participants might not learn about an appointment until the day of made it even harder to manage work responsibilities. Participant 10 noted that: “I had to work a lot from home because I had to go to clinic almost every day. My mind was somewhere else and I couldn’t do anything. So, my performance was affected a lot. … It’s hard to concentrate on a career when I have so much going on”.

Similarly, participant 13 stated that “while you are going through all the procedures work is almost impossible”, while participant 14 noted that “I certainly feel it has impacted work, and it makes life more stressful both at work and at home”. In an effort to decrease stress, a few participants quit their jobs or dropped out of graduate school. This was the case for participant 15, who stated that infertility “played a huge role in my decision to leave my job …”.

“Infertility is very lonely” (Participant 10) (Sub-themes: Secrecy and misunderstood)

Participants frequently expressed a sense of social isolation, saying it would be nice to have someone to talk to about their challenges and experiences. Participant 17 noted that, “the biggest barrier has been the social isolation. I used to have a lot of friends, still do. But social relationships were a big part of my life”. Participant 16 stated one of the hardest things about infertility is “that nobody gets it. You try reaching out and people say something offensive or insensitive, and you cut them off from that part of your life. That in turn makes you not want to reach out to others. It’s very isolating”. This included often having trouble relating to others, whether friends, family, or co-workers, who have children. As participant 14 noted, “it’s very isolating and you feel very alone because people in the general public don’t get it, don’t understand how all-consuming it is in your life”. Participant 10 echoed the sentiments of many participants when she stated, “infertility is very lonely”.

### Secrecy:

Participants expressed that they often felt the need to keep their infertility struggles secret. They see it a taboo subject that people do not want to talk about, and feel that there is a stigma attached to those with fertility struggles. Participant 3 explained that “there is a stigma that I don’t think there should be about infertility”, and participant 5 noted that “it’s something I avoid because people don’t understand or try to understand”. Furthermore, participant 8 noted that “I do keep people at arm’s length for personal protection”. For some participants, keeping it a secret was the hardest thing associated with the experience. They often had trouble determining who to open up to, and how to respond to questions related to when they were going to have children. As participant 20 explains, “people ask about when we will have it [children], and we don’t want to explain to them the truth”. Participant 15 stated that “You need to push them [friends] away a little bit”. The couple experiencing infertility may not go out as much as they use to, finding it easier not to talk than to try and keep their infertility struggles a secret. This can be made even more difficult and “brutal” when a friend or family member gets pregnant, or when they are questioned and teased about when they are going to have a baby. Such feeling led to many participants choosing not to reveal their infertility struggle with their parents, which made it even harder at times to communicate and be open with them. Participant 14 noted that: “In general, going through the whole fertility process has made us more isolated. I don’t reach out as much as I used to, or there are times when you can’t really go out and do things because you are on medication cycles. When maybe I would have liked to have gotten together, I couldn’t because of the medication cycle and having to be home. I would say I don’t spend as much time with my friends as I used to. Trying to keep information quiet can get exhausting, so sometimes it’s easier to just not talk to them”.

### Misunderstood:

Participants noted that it can be hard to connect with others, feeling like people will not understand their struggles. Participant 19 expressed that: “Unless you have been through it, you just can’t understand it. … I just shut down. I felt people couldn’t understand it, and I didn’t have the energy to explain it. I didn’t want to talk about it. There was nothing anyone could say that would make me feel better about it. It was better for me to hide in my cocoon”.

Similarly, participant 12 reported that, “most people have no idea what infertility really looks like, or IVF treatment”, while participant 3 noted that “it is kind of hard to be different”, and participant 5 stated that people “discount your feelings. … They don’t take into consideration that it hurts. … They don’t realize that it hurts your feelings”. Participant 8 reported that one of the hardest things is “Fitting in. I’m the only one [of her friends] who doesn’t have kids by now”. This was echoed by participant 11 noted that the existing friendships of she and her husband has been impacted.

because they don’t know how to be there for us. … I think what it is that you don’t feel like you can be your whole self, or your true self. People only know this surface part of you because you either don’t feel comfortable that you can tell them without it getting weird, or then you do tell them and you are almost even more disappointed because they change the subject or don’t want to talk about it. It feels like rejection of who you are and what you are dealing with.

Furthermore, participant 10 noted that: “You don’t really talk about it, so I feel like a lot of times, I mention it to some friends, but I feel like if people don’t experience it they don’t really understand it. You don’t feel close to them anymore”.

Participant 12 also noted that one of the hardest things about experiencing infertility is

Peoples lack of understanding of it, or lack of empathy. You have people that don’t understand why you wouldn’t want to attend a baby shower when you go through this, or don’t understand why you don’t want to hold a child. Or just thinking of my sister not sending us as many pictures or not including my parents a little more to see their grandkids, knowing that maybe their only grandchildren [live in another state]. … Peoples empathy or understanding of what infertility really looks like on a daily basis.

Participants also did not want to burden others by talking about their infertility challenges. This has led to participants losing friends and feeling discounted and misunderstood. They experienced a lack of understanding and empathy from others who cannot understand what they are going through as the societal pressure to have children is pushed on them. One participant explained that no one seems to get it, and that when she has reached out to others they are offensive or insensitive, pushing their choices on her. This has caused her to cut them out of her life and to not want to reach out to others. Participant 18 expressed, “I try to separate myself so it’s not as painful”. A reason for this, participant 11 explained, might be “because they don’t know how to be there for us. …

It [sometimes] feels like rejection of who you are and what you are dealing with”. As a result, participant 8 reported “I do keep people at arm’s length for personal protection”.

## Discussion

Findings show that experiencing infertility can have a tremendous impact on an individual’s daily life and roles. The expenses and time commitment of exploring fertility treatments make it difficult for individuals to continue on with their daily routines and occupations, including self-care, work, and leisure pursuits. This can require one to prioritize and work on balancing all areas of their lives. How one prioritizes is unique to each individual, and can include spending more or less time on self-care, leisure, and career activities. Prioritizing can impact relationships and roles as well. The lack of fulfilling a desired role that infertility leads to can result in various emotional challenges (11). Due to the emotional stress, potential emotional triggers, and the various challenges associated with infertility and infertility treatments, individuals might decide to forgo engaging in social activities with friends and family members. This can lead to losing touch and connections with people.

Another reason for decreasing social interactions can be the negative, unsympathetic response others may have when discussing infertility. This can be due to simply not knowing what to say, or not being aware of what an emotional and sensitive issue infertility can be. The desire to withdraw socially, and feeling misunderstood by others, is consistent with previous literature (1, 2). Overall, the unpredictability of fertility treatments, along with the emotional toll infertility can take, can lead to a sense of loss of control (2). The challenges that participants in the current study expressed can lead to a decrease in overall well-being and impact all areas of life, which is consistent with previous literature (12–14). Of note, some participants in the current study as well as in previous research studies have found that engaging in activities with the children of friends or family members was found to be a way to be involved in the lives of children (15).

As infertility is often seen as a taboo subject, many keep their infertility a secret. The stigmatization of infertility and the need to keep infertility a secret that participants expressed has also been noted in previous literature (12, 15–17). This means they do not get the support that they might need from others. Even when they did discuss their challenges with others, participants often felt misunderstood. This could possibly be related to the notion that infertility has largely been found to be a social construct (16). The results of decreased motivation are connected with support seeking of infertile couples from others. As it has the potential to impact every area of their life, individuals experiencing infertility need to have more resources and support available. Previous research has found that women experiencing infertility tend to experience more stress and life disruption than males experiencing infertility, so they may need extra support (18). This can include resources on how to effectively communicate with spouses/partners and other family members, strategies on how to deal with emotional triggers such as seeing a pregnancy announcement on social media, and exploring alternative ways to have a family.

Overall, infertility was found to alter participants’ daily life. They had to make adjustments to their daily routine for a variety of reasons, including the time commitment and physical effects of infertility treatments. These, in addition to the emotional aspect of infertility, such as depression, resulted in many participants having decreased desire and drive to complete daily tasks such as cleaning and self-care. Their leisure pursuits and careers were also frequently decreased and altered. They often felt like they were in survival mode, doing only what was necessary. Furthermore, the challenging emotional impact infertility can have on women was the decrease of their desire to participate in social gatherings with friends and family, as they may frequently feel misunderstood. This impacted their ability to fulfill their roles as friend, daughter, sister, and aunt.

It is important to note that each individual’s experience is different, and as a result each individual will require different types of resources and support. This has been discussed in the literature, where individuals experiencing infertility have expressed the desire to receive client-centered, individualized care (11). Some might struggle more with the physical aspects of infertility, such as the effects of infertility treatments, while others might struggle with time management or depression. Resources should also be provided to help those experiencing infertility understand that what they are feeling and experiencing is normal and okay, and to feel comfortable talking about it with others, including friends or family members, or professionals. Health care professionals also need to be more sensitive to the needs and challenging emotions individuals experiencing infertility may have and offer the appropriate support and resources. This includes providing education on other methods to have a child, such as medical interventions or through adoption. This was a need found consistent with other research (15).

Finally, it is essential for health care professionals and others to know that each individual experiencing infertility is unique, and that you may never know the potential outcomes. For example, one participant who had been told she could never get pregnant became pregnant during the time between her first and second interview. She was able to carry the baby to full-term. Participants noted that their interactions with healthcare professionals sometimes influenced their emotions. It can be helpful when the healthcare professional takes the time to listen to the individual, address their needs, and understand their emotions and situation. As participant 4 reported, “it’s a totally different conversation depending on who that person is. … Everyone deals with it [infertility] differently”.

### Limitations:

The fact that only women were interviewed in this study was one of the limitations. Males experiencing infertility might have different experiences and perceptions. In addition, some participants (Such as those with Turner Syndrome) knew at a younger age, even before trying to have children, that they would most likely not be able to have children. This may have impacted their view of infertility. Furthermore, though participants were from all over the United States, participants were largely Caucasian. There was limited cultural and racial diversity. As this was a qualitative research study, the aim was not to generalize the results. Finally, while there did not appear to be a difference, the current study did not focus on the impact duration of infertility may have in the experience of the participant’s occupations and roles.

### Suggestions:

Interviewing couples who are experiencing infertility would be helpful to get a more holistic view of the experience. Exploring the impact of culture on the infertility experience would also be worthwhile. Various healthcare professionals could also be interviewed to obtain their perception of the experience, knowledge of the resources available, and the resources and support they offer.

## Conclusion

Infertility has the potential to impact every area of a woman’s life. Those experiencing infertility need more resources and support to navigate their journey, including emotional and physical needs. The emotional impact infertility may have on women, in addition to the physical and time constraints involved with pursuing fertility treatments, frequently resulted in decreased occupational engagement and fulfillment of roles as spouse or friend.
